# Homicide, Inequality, and Climate: Untangling the Relationships

**DOI:** 10.3389/fpsyg.2021.697126

**Published:** 2021-08-11

**Authors:** Lawrence A. Kuznar, Jeffrey Day

**Affiliations:** ^1^Department of Anthropology and Sociology, Purdue University, Fort Wayne, IN, United States; ^2^National Security Innovations, Inc., Boston, MA, United States

**Keywords:** homicide, inequality, risk sensitivity, temperature, climate, class

## Abstract

Researchers debate the causal connections between homicide, inequality, and temperature. This study examines these relationships globally based on country-level data. A new measure of inequality is introduced that provides a more granular measure of inequality patterns than commonly used metrics. The approach allows estimation of risk sensitive decision-making that helps to explain how class impacts violence under different climate conditions. The results indicate that homicide rates are higher when poorer segments of populations are disproportionately influenced by temperature, middle class segments are influenced by inequality, and the wealthy are influenced by middle and impoverished class dynamics.

## Introduction

The positive association between inequality and homicide is well-established (Blau and Blau, 1982; Bailey, 1984; Wilson and Daly, 1997; Daly, 2016) and holds at social scales from communities all the way to countries (Levitt, 1999; Messner et al., 2002; Ouimet, 2012; Rufrancos et al., 2013; Harris and Vermaak, 2015; Daly, 2016; Di Matteo and Petrunia, 2019). As a matter of definitional clarity, we adopt the U.N. definition of homicide as intentional homicide, which is unlawful death purposefully inflicted on a person by another person, excluding unintentional homicides and deaths due to armed conflict (UNODC, 2019).

Despite the pervasiveness of the relationship between inequality and homicide, scientific consensus to explain it remains elusive. Kelly (2000) argues that inequality creates stresses and erodes social norms among the poor, making homicide more likely. Using the U.N. Human Development Index (HDI), Ouimet (2012) demonstrates that inequality is associated with homicide in countries with medium to high human development. In impoverished low HDI countries, economic factors such as poverty and inequality interact with high proportion of youth, decreasing the effectiveness of the criminal justice system, which in turn leads to increased homicide. Daly (2016) argues that the concentration of wealth and status among wealthy males causes poorer males to compete more lethally over their dwindling supply of social status.

Other researchers have proposed that the association between inequality and homicide is spurious, noting that homicide rates tend to be higher in hotter climates and on hotter days (Cheatwood, 1995; Anderson et al., 1997; Mishra, 2015; Heilmann and Kahn, 2019). As with inequality, there is debate over the causal mechanisms that underly this association (Miles-Novelo and Anderson, 2019). Some researchers argue that it is a function of heat stress, which weakens impulse control (Anderson et al., 1997). For instance, recent research indicates that heat interferes with serotonin reception to reduce inhibition, making homicide more likely (Tiihonen et al., 2017). Alternatively, the routine activity hypothesis proposes that homicide is more common in warm weather simply due to the fact that people intermingle more in warm weather, providing more opportunities for violence, and spend more time outdoors where they are less protected, placing themselves at greater risk (Cheatwood, 1995; Rotton and Cohn, 2003; Miles-Novelo and Anderson, 2019). Coccia (2018) importantly demonstrates that there is a strong correlation between hot climate and inequality that confounds the relationship between homicide, inequality, and temperature.

The association between heat and homicide is reinforced at deep historic and prehistoric time scales (Hsiang et al., 2013), and growing climatic warming and associated climate change has created fears that violence will increase (Rotton and Cohn, 2003; Mares and Moffett, 2016; Van Lange et al., 2016; Miles-Novelo and Anderson, 2019). Anderson et al. (1997) pioneering study of the 50 largest U.S. metropolitan areas established a strong positive relationship between temperature and homicide through time. In another early study, Rotton and Cohn (2003) found that temperature was associated with assault, rape, robbery, and burglary through time, but not homicide in a state-level study of the United States, and in a study of U.S. counties, found that temperature had a strong bivariate effect on all major categories of crime.

Recent empirical studies have largely corroborated the association of climate change and homicide, but the results are mixed and nuanced. Climate change appears to have an indirect effect on homicide, mediated by other factors. For instance, Barlett et al. (2020) provide a path model based on country-level correlations from 1961 to 2015 that connects global warming to extreme weather events that threaten clean water supplies, which they infer creates resource stresses that motivate homicide. Similarly, Peñaherrera-Aguirre et al. (2019) conducted a 25-year moving average study demonstrating that climate change exacerbates resource competition and inequality, which is mediated through proposed evolutionary influences based on latitude, cultural norms, and climate change. Furthermore, some studies find mixed or no association between temperature and homicide. A study of nine U.S. cities from 2007 to 2017 found that the positive association between temperature and homicide held only for Chicago and New York (Xu et al., 2020). Finally, a study of New York and London covering the years 1895–2015 found that including per capita GDP in a statistical model eliminated the temperature effect on homicide, and correcting for serial autocorrelation eliminated all relationships (Lynch et al., 2020). These recent studies indicate that further research is necessary to establish a clear link between climate change, notably global warming, and increased homicide.

In this paper, the relationships between homicide, inequality, inequality-driven risk sensitivity, and temperature are explored on a global country-level data set from 1960 to 2019. Inequality is examined with several different metrics. The Gini coefficient measures overall patterns of inequality, the percent population below the poverty line measures the effects of absolute poverty, and a new measure of inequality based on wealth and status distributions provides an examination of these relationships for poor, middle class, and wealthy segments of society. The fundamental finding is that inequality is the prime driver of homicide rather than temperature, although inequality-driven risk sensitivities of poor, middle-class, and wealthy segments of society interact with temperature. These interactions have potential policy implications and deserve further scrutiny.

## Inequality, Social Status, And Risk Sensitivity

The association of inequality and homicide suggests further investigation regarding how inequality and class differences are related to lethal violence. Friedman and Savage (1948) suggested that unequal distributions of wealth and social status impact individuals' utility functions (satisfaction derived from status), which in turn influence an individual's willingness to take or avoid risks. Despite their focus on private individual subjective utility functions, their copious footnotes, almost equal to the text of the article, provide data and arguments suggesting that the publicly observable distribution of wealth influences individuals' utility functions and sensitivity to risk. The use of violence, especially among peer-competitors who have the same access to the means of violence, is by definition highly risky behavior and so should be influenced by wealth and status differences (Wilson and Daly, 1997; Kuznar, 2007; Daly, 2016).

**Figure 1:**
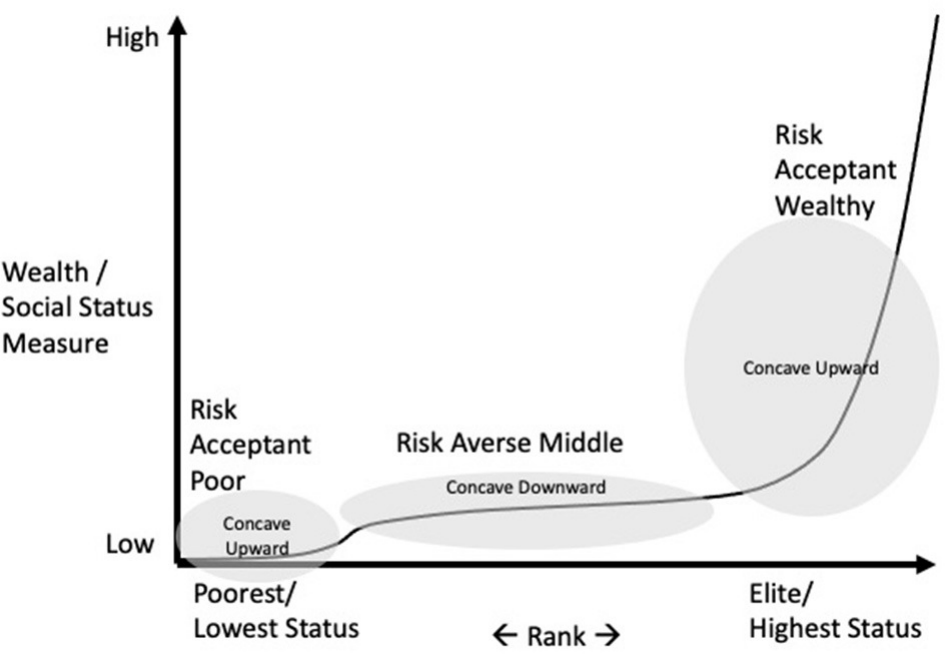
Wealth distribution, class, and risk sensitivity.

Inequality is measured many ways including percentage of wealth owned by the top *x* percent, percent of a population living in poverty, or the commonly used Gini Coefficient, which is the difference between the Lorenz curve, defined by percent wealth of each percentile of a society, and the line of total equality, in which each percentile of society shares equally in society's wealth (see Kovacevic, 2010 for a full explanation). Each of these measures provides insight into how wealth is distributed in a society, but each obscures variation in inequality between classes. An alternative way of describing inequality records the wealth of each percentile of society against the rank of each percentile in wealth, as suggested by Friedman and Savage (1948). This reveals abrupt increases of wealth as one moves from the poorest to the wealthiest ranks in a society; wealth class boundaries are defined by relatively sharp increases in the curve. Figure 1 represents a wealth distribution curve typical of most societies; it has a low tail for the very poor, followed by a sharp increase in wealth that is fairly level and defines a middle class, which is then followed by an extremely sharp increase that continues to the wealthiest individuals in a society. Mathematically, this curve has an initially concave upward section (the poor), followed by a concave downward segment (a middle class), followed by a strongly concave upward section (the wealthy). The curve reflects the fact that wealth is typically concentrated at the top of most societies, and research demonstrates that this pattern is found in societies as varied as small tribes to ancient kingdoms, modern states, and even the entire world economy (Kuznar, 2001, 2002, 2007; Lewis, 2004).

The practical utility of wealth is obvious; it can be used to purchase goods and services people need and desire. However, wealth also signals social status. Tokens such as metal or shell armbands in ancient and current tribal societies are classic examples (Mauss, 1967; Malinowski, 1985). Industrial societies are no different. Conspicuous consumption (Veblen, 1994) by the wealthy is abundantly present in modern society; they build elaborate mansions and buy luxury cars whose cost far exceeds what is necessary to satisfy basic needs of shelter and transportation. The wealthy are not the only people interested in tokens of status. A study of social media discussions among the U.S. general public found a positive correlation between inequality and a desire for status goods (Walasek and Brown, 2015). Wealth has much greater significance than purchasing power, it signals one's position in society and consequently one's social worth and status.

Wealth has material and social value and therefore one would expect people to compete for it. However, not everyone is equally motivated to compete. People whose utility functions are concave downward are expected to avoid risk and competition because more status can be lost vs. what could be gained; risk aversion is a widespread human tendency (Bernoulli, 1954; Cashdan, 1985). One would expect people to accept risks to achieve status when potential gains can exceed potential losses, represented by the concave upward sections of Figure 1 (Friedman and Savage, 1948; Markowitz, 1952). This pattern of risk-taking behavior has been confirmed across an incredibly wide array of cultures including hunting and gathering bands, tribes, ancient kingdoms and modern states (Pryor, 1976; Kuznar, 2001, 2002). Risk taking to gain status can take many forms. Legal and socially accepted forms of risk taking include investing in the stock market or starting a legal business. However, people may engage in unsanctioned or illicit forms of risk taking as well. Violently challenging rivals for status is by definition risky. For instance, political science research has identified relative differences in wealth as a core motive for lethal political risk taking, including revolutions (Gurr and Moore, 1997; Besancon, 2005), terrorism (O'Neill, 2000; Kuznar, 2007; Kuznar and Lutz, 2007), and mass protest movements (Midlarsky, 1988).

## Methods and Data

Pratt (1964) provided a measure of risk sensitivity for an individual at different levels of wealth. Subsequent work by Arrow (1974) reinforced his research and it is known as the Arrow-Pratt measure of risk aversion. The measure is calculated as:

Equation 1. Arrow-Pratt Measure of Risk Aversion.

$$r\left(x\right)=-\left(\frac{U{\left(x\right)}^{\prime \prime }}{U{\left(x\right)}^{\prime }}\right),$$

where *U(x)* is a utility function that measures satisfaction for differing levels of wealth, *x*.

The measure can range from –∞ to +∞; negative values indicate the degree of risk acceptance and positive values indicate the degree of risk aversion. To the extent that wealth is a measure of social status, the distribution of wealth therefore creates a function for the utility of social status, which was implied in the original proposition for measuring utility by Friedman and Savage (1948). Therefore, by fitting a curve to a wealth distribution, one can measure wealth's utility for conveying social status, and the Arrow-Pratt measure can be applied to determine the risk sensitivity of an individual at any level of wealth. The function fitted to a wealth distribution is called the expo-sigmoid function (Kuznar, 2007) because typical wealth distributions are generally exponential (wealth concentrates at the top), but exhibit sigmoid (S-shaped) oscillations that define wealth classes (Figure 1). The function is:

Equation 2. Expo-sigmoid utility function.

$$S\left(rank\right)=e^{a+b\left(rank\right)+csin\left(rank\;\right)+dcos(rank)},$$

where *S* is the expo-sigmoid fit to the wealth distribution, and *rank* is the rank from poorest to wealthiest in the society. A full description of the curve fitting method is presented in Kuznar (2007).

The World Bank provides publicly available data on homicide, Gini coefficients, percent population below the poverty line (a measure of absolute poverty), and wealth distributions for all countries from 1960 to 2019, enabling measurement of wealth distributions and Arrow-Pratt measures and testing the relationships between all of these variables. These data were used to create wealth distribution curves for the 173 countries with 2019 populations over 300,000, which excludes small tropical Island states who are often outliers in a variety of social measures. Expo-sigmoid curves were fit to their wealth distributions and Arrow-Pratt measures were calculated for each centile. The Arrow-Pratt measure was averaged over these centiles, providing an overall measure of a country's risk sensitivity. Nearly every country exhibited an upward concave poor class, a concave downward middle class, and the wealthy were uniformly strongly concave upward in every case. In order to provide a finer-grained analysis of risk sensitivity and homicide in a society, the average Arrow-Pratt measure was calculated for each of these population segments.

The World Bank provides homicide rates from the U.N. Office on Drugs and Crime International Statistics, which is recognized as a source of reliable homicide data (Ouimet, 2012). The distribution of the homicide rate by country is heavily skewed toward the lower end, consequently we used its natural log to make it more “normal” and thus more appropriate for parametric statistical analysis. Due to missing data, the sample consisted of 679 country-years. Data imputation methods were employed to fill in missing data, but the resulting analyses provided the same qualitative results as the raw data. Therefore, only the original 679 observations were used in order to remain as true to the original data as possible.

Countries have different histories of homicide due to serial autocorrelation (rates in 1 year tend to carry over to the next), and varying levels of social control, policing, and cultural norms surrounding violence. The logged homicide data show break-points between low homicide countries (LnHomicide < −0.23, raw homicide rate < 0.8/100,000, number of cases = 124), high homicide countries (LnHomicide > 2.46, raw homicide rate > 11.7/100,000, number of cases = 98), and medium homicide countries in-between (number of cases = 457). These country-specific effects should be taken into account; panel regression is a common method that takes into account within-group variance on key variables within a linear model (Wooldridge, 2010). Therefore, panel regressions were conducted using panels of low, medium, and high homicide countries.

Because the inequality metrics were highly correlated (Table 1), separate models were run for each of the inequality metrics (Gini coefficient, absolute poverty, and the risk sensitivity measures) while controlling for temperature. This allowed each of the inequality metrics to compete statistically with temperature in the association with homicide, avoiding multicollinearity between the inequality metrics and providing insights into (a) the relative importance of inequality and temperature, and (b) varied insights provided by the different inequality measures.

**Table 1:** Correlations between key variables.

	**LnHomicide**	**Temperature**	**Gini**	**Absolute poverty**	**Avg risk sensitivity**	**Risk sensitivity of poor**	**Middle class risk sensitivity**	**Wealthy risk sensitivity**
LnHomicide		0.267^***^	0.704^***^	0.530^***^	−0.625^***^	−0.475^***^	−0.503^***^	0.074^*^
Temperature			0.570^***^	0.478^***^	−0.471^***^	−0.309^***^	−0.426^***^	0.091^**^
Gini				0.598^***^	−0.913^***^	−0.731^***^	−0.680^***^	0.243^***^
Absolute poverty					−0.545^***^	−0.357^***^	−0.388^***^	0.097^**^
Avg risk sensitivity						0.903^***^	0.456^***^	−0.072^***^
Risk sensitivity of poor							0.370^***^	−0.094^**^
Middle class risk sensitivity								−0.445^***^

* *Statistically significant at the 0.05 level*,

** *significant at p ≤ 0.01 level*,

*** *p < 0.001*.

## Results

Table 1 presents the raw Pearson correlations between the independent variables based on inequality, poverty, and temperature, and the dependent variable homicide. These data confirm the first order effects of each of the independent variables on homicide.

However, the correlations between the independent variables are very high, requiring an account of the relative effects of each independent variable in relation to one another. Ordinary least squares models were employed, using standardized coefficients to examine the statistical significance and relative strengths of the relations between homicide and the inequality, controlling for temperature (Table 2). Separate models were run for each inequality or poverty measure, and always included temperature in order to test for the relative effects of inequality vs. temperature.

**Table 2:** Comparison of linear models of the effects of inequality and temperature on Ln homicide rate.

**Attribute**	**Model 1**	**Model 2**	**Model 3**	**Model 4**	**Model 5**	**Model 6**
	**Gini**	**Absolute poverty**	**Avg risk sensitivity**	**Poor risk sensitivity**	**Middle-class risk sensitivity**	**Wealthy risk sensitivity**
**Temperature**						
B^a^ (Standard error)	−0.007 ns (0.0043)	0.072* (0.029)	0.084** (0.030)	0.134*** (0.030)	0.052 ns (0.031)	0.138*** (0.029)
**Gini**						
B (Standard error)	0.348*** (0.043)					
**Absolute Poverty**						
B (Standard error)		0.278*** (0.034)				
**Average risk sensitivity**						
B (Standard error)			−0.182*** (0.042)			
**Risk sensitivity of the poor**						
B (Standard error)				−0.043 ns (0.033)		
**Middle class sensitivity**						
B (Standard error)					−0.231*** (0.032)	
**Wealth risk sensitivity**						
B (Standard error)						0.108** (0.035)
*R* ^2^	0.114^***^	0.113^***^	0.054^***^	0.030^***^	0.097^***^	0.041^***^
*F* (df = 2, 674)	45.79^***^	45.32^***^	21.23^***^	12.45^***^	38.35^***^	16.60^***^

Ns, not statistically significant, *significant at the 0.05 level, **significant at p ≤ 0.01 level,

*** *p < 0.001*.

a *Data were standardized so that all coefficients were directly comparable in magnitude, therefore coefficients are standardized*.

The Gini and Absolute Poverty models have the highest adjusted *R*^2^ values (0.114, 0.113, respectively), indicating that they perform best. They demonstrate highly statistically significant and substantially higher standardized coefficients for inequality than temperature; absolute poverty has over four times the effect on homicide and the Gini coefficient is 50 times more associated with homicide than temperature. By these measures of inequality, temperature loses its association with homicide world-wide, and inequality appears to be the real driver. Average risk sensitivity is also more associated with homicide than temperature, about twice as much so, but the independent effect of temperature is also statistically significantly related to homicide, although the model explains less than half the variance of the Gini and absolute poverty models.

The value of taking a risk sensitivity approach emerges when examining how inequality and temperature operate within classes. The risk sensitivity of the poor is not statistically related to homicide rates, but temperature statistically is and by three times as much as risk sensitivity. The opposite relation holds for the middle class; as they become more risk acceptant homicide rates increase strongly (4.4 times stronger) and temperature is not statistically related. The wealthy present a counter-intuitive result for inequality. As their risk acceptance decreases, homicide rates increase, and the effect of temperature is slightly stronger.

## Discussion

Overall, in this study temperature performs poorly when it has to compete with measures of inequality for explaining homicide as previously demonstrated by Coccia (2018). However, the risk sensitivity measure, disaggregated by social class, provides insights into the conditions when temperature may be an important factor.

The model based on the risk sensitivity of the poor shows no statistically significant association between homicide and their risk sensitivity, but a strong association with temperature. This indicates that relative poverty may expose people more to the effects of temperature, which can lead to homicidal behavior. For instance, Cheatwood (1995) notes that access to technological means of mitigating heat may lower the likelihood for committing homicide, the very means the poor typically lack. Furthermore, Heilmann and Kahn (2019) argue that extreme heat decreases policing efforts and therefore social control mechanisms in poor neighborhoods, which contributes to higher homicide rates in impoverished areas. Contrasting the results for the poor, homicide rates are strongly influenced by increased risk acceptance among the middle-class but temperature has no discernable effect. This could be due to the fact that middle class people most likely have means with which to mitigate the effects of increased temperature, leaving risk acceptance as the dominant influence on homicide rates. Additionally, a strongly risk averse middle class may lobby for and support more aggressive social control in order to protect themselves and their assets from violence, and as their risk aversion decreases they may be less supportive of these measures, in turn raising homicide rates.

The relationships between homicide, inequality, and temperature among the wealthy are more challenging to explain. As the wealthy become more risk averse and as temperature rises, homicide rates increase. This may be a function of the fact that poorer countries tend to be located in hotter regions of the world (Coccia, 2018). It is possible that the poorer a country is, the less wealth differences exist between the wealth, which would decrease their risk acceptance. As demonstrated in the risk acceptant poor model, the poor are particularly susceptible to the effects of heat because of their absolute poverty, raising overall homicide rates for a poor country in a hot region. Other homicide researchers shed further light on these results. Coccia (2018) argues that during European colonization, hot climates where Europeans were exposed to disease mortality led to the development of extractive economies designed to bring resources back to Europe, as Europeans sought ways to avoid living in these uncomfortable and for them unhealthy climates. The extractive colonial economies therefore left impoverished underdeveloped economies more prone to homicide in the world's hot regions. Alternatively, Park et al. (2018) used survey data on 690,000 households in 52 countries and found that in hot countries, temperature was associated with lower household income. They argue that exposure to heat stress lowers the economic productivity of the poor whereas the wealthy are able to move to cooler parts of the country, exacerbating wealth differences in developing countries. However, decreased productivity of laborers decreases overall GDP for that country. In all cases, researchers are careful to stress that they can comment only on associations since the specific causal mechanisms are as yet unknown. However, these results reinforce the associations between poverty and homicide in warmer regions of the world, regardless of the risk sensitivity of the wealthy.

This research points to several implications for homicide reduction. First, simple equations between homicide and inequality or temperature are not nuanced enough to support effective policy decisions concerning homicide. Inequality is the overarching dominant influencer of homicide, but the impact of temperature appears to be related to class. The data presented in this article indicate that, contrary to Daly (2016), if the poor risk violence to get ahead they will neither gain nor lose much in social status. Therefore, the poor have little rational reason to use violence to get ahead because the cards are stacked against them. Increased temperatures, however, appear to have a disproportional impact on the poor, leading to increased interpersonal homicide. Addressing their basic needs for shelter and relief from heat may a more effective way to decrease homicide rates. The middle class is typically the most peaceful segment of society; they fear loss and so are less likely to take risks and compete, especially in violent ways. However, this study's results indicate that when their status is threatened, that is when their risk aversion decreases, homicide rates can strongly increase. This result invokes loss aversion, in which the fear of loss switches a normally risk averse decision maker to one acceptant of risk taking (Kahneman and Tversky, 2000). Whether this is because they become more violent or because their support for social control decreases with their risk acceptance is unclear and requires further study. Finally, the wealthy present a challenging case. The patterns between wealthy risk sensitivity and homicide appear to represent a complex interaction between their level of risk sensitivity and its impact on middle and lower classes.

The results of this study suggest further research. Exactly who kills whom is unclear in available country-level statistics. Data on the class of perpetrators and victims is needed to test the relationships between class, risk sensitivity, and homicide suggested by this analysis. Furthermore, the indirect mechanisms that might link risk sensitivity levels in one class to homicides in another need to be explored. The relationship between elite inequality and homicide also needs to be explored further. People of means not only have alternate and non-violent means of competing, but they also compete in lethal ways that are not classified as interpersonal homicide, as in coups and leading rebellions (Brinton, 1964; Braithwaite et al., 2019), which disproportionately take place in warm weather countries of the developing world. Therefore, research into the linkages between inequality, temperature, and political violence should also be explored. Finally, much work needs to be done to explore other causal factors that operate on the country level. Inequality and temperature are clearly pieces of the puzzle, but not the whole picture.

## Data Availability Statement

Publicly available datasets were analyzed in this study. This data can be found at: Harvard Dataverse https://doi.org/10.7910/DVN/OWYI1B.

## Author Contributions

LK was the primary author of this article and developed the theory and methodological approach. JD provided technical support for the data analysis. All authors contributed to the article and approved the submitted version.

## Conflict of Interest

Both authors were employed by the company NSI, Inc. The authors declare that the research was conducted in the absence of any commercial or financial relationships that could be construed as a potential conflict of interest. Furthermore, the views of the authors do not represent the official views of either Purdue University Fort Wayne or NSI, Inc.

## Publisher's Note

All claims expressed in this article are solely those of the authors and do not necessarily represent those of their affiliated organizations, or those of the publisher, the editors and the reviewers. Any product that may be evaluated in this article, or claim that may be made by its manufacturer, is not guaranteed or endorsed by the publisher.
